# MEKK3-MEK5-ERK5 signaling promotes mitochondrial degradation

**DOI:** 10.1038/s41420-020-00342-7

**Published:** 2020-10-20

**Authors:** Jane E. Craig, Joseph N. Miller, Raju R. Rayavarapu, Zhenya Hong, Gamze B. Bulut, Wei Zhuang, Sadie Miki Sakurada, Jamshid Temirov, Jonathan A. Low, Taosheng Chen, Shondra M. Pruett-Miller, Lily Jun-shen Huang, Malia B. Potts

**Affiliations:** 1 Department of Cell and Molecular Biology, St. Jude Children’s Research Hospital, Memphis, Tennessee 38105 USA; 2 Integrated Biomedical Sciences Program, University of Tennessee Health Science Center, Memphis, Tennessee 38163 USA; 3 Department of Cell Biology, The University of Texas Southwestern Medical Center at Dallas, Dallas, Texas 75390 USA; 4 Department of Hematology, Tongji Hospital, Tongji Medical College, Huazhong University of Science and Technology, Wuhan, China; 5 Department of Chemical Biology and Therapeutics, St. Jude Children’s Research Hospital, Memphis, Tennessee 38105 USA

**Keywords:** Mitophagy, Stress signalling

## Abstract

Mitochondria are vital organelles that coordinate cellular energy homeostasis and have important roles in cell death. Therefore, the removal of damaged or excessive mitochondria is critical for maintaining proper cellular function. The PINK1-Parkin pathway removes acutely damaged mitochondria through a well-characterized mitophagy pathway, but basal mitochondrial turnover occurs via distinct and less well-understood mechanisms. Here we report that the MEKK3-MEK5-ERK5 kinase cascade is required for mitochondrial degradation in the absence of exogenous damage. We demonstrate that genetic or pharmacological inhibition of the MEKK3-MEK5-ERK5 pathway increases mitochondrial content by reducing lysosome-mediated degradation of mitochondria under basal conditions. We show that the MEKK3-MEK5-ERK5 pathway plays a selective role in basal mitochondrial degradation but is not required for non-selective bulk autophagy, damage-induced mitophagy, or restraint of mitochondrial biogenesis. This illuminates the MEKK3-MEK5-ERK5 pathway as a positive regulator of mitochondrial degradation that acts independently of exogenous mitochondrial stressors.

## Introduction

Maintaining a population of healthy mitochondria is vital for proper development and is necessary to preserve tissue and organ function in response to stress conditions^1^. Mitochondria are well known for their importance in metabolism and energy production, but they are also a major source of reactive oxygen species (ROS), cytosolic DNA, and pro-apoptotic proteins^2–5^. Therefore, it is important to maintain sufficient quantities of healthy mitochondria to meet current metabolic demand, but dangerous to carry an excessive surplus of these organelles. Damaged or dysfunctional mitochondria tend to generate higher levels of ROS and cytosolic DNA compared to healthy mitochondria^2,3,6^. Exposure to high levels of ROS can cause DNA mutations in both nuclear and mitochondrial DNA and can induce protein misfolding throughout the cell^2,3^. Mutations in mitochondrial DNA further decrease the health of the mitochondrial population and promote additional ROS production^7–9^. Thus, to maintain homeostasis, the cell has evolved complex systems for the quality control of mitochondria^10^. These systems are designed to balance the elimination of dysfunctional or superfluous mitochondria with mitochondrial biogenesis^1^. There are certain situations in which mitochondrial content changes even in the absence of damage, such as during differentiation of highly specialized cell types or induction of a metabolic shift towards a glycolytic phenotype or an oxidative phosphorylation phenotype^11–14^.

A major mechanism of mitochondrial degradation is known as mitophagy, a form of selective autophagy^10^. Autophagy is an evolutionarily conserved process that uses double membrane vesicles known as autophagosomes to sequester cellular components such as damaged or excessive organelles and protein aggregates^15^. Autophagosomes then fuse with highly acidic lysosomes that contain hydrolases, which degrade the components inside^15,16^. Mitophagy follows the same steps as autophagy, but the autophagosome selectively forms around mitochondria that have been tagged for degradation by selective autophagy receptors such as NIX, OPTN, or p62/SQSTM1^10^. These proteins function by binding both the cargo and ATG8-family proteins (commonly referred to as “LC3” proteins) that decorate the forming autophagosome^17^.

Mitophagy plays important roles in development and in disease prevention. Insufficient mitochondrial clearance impairs erythroid maturation and can result in anemia^18^. Removal of paternal mitochondria after zygote formation increases fitness, as mitochondrial DNA is at increased risk of ROS damage during the process of spermatogenesis and fertilization^19^. Parkinson’s disease, the second most common neurodegenerative disorder, is characterized by dysfunctional mitochondria and compromised mitophagy within the affected neurons^20–22^. In addition, dysfunctional mitochondria can contribute to the metabolic reprogramming of cancer cells^23^. Understanding how novel regulatory pathways contribute to the maintenance of mitochondrial homeostasis in different tissues could provide insights into the development of targeted treatments when these systems fail in disease.

Early insights into the mechanistic basis of mitochondrial degradation arose from studies in yeast, but how mitochondrial degradation is controlled in multicellular organisms such as humans is not yet fully understood^24–26^. Pink1-Parkin is the most well studied mitophagy regulatory pathway in animals. Although it has been very well characterized, most studies depend on ectopic Parkin overexpression and mitochondrial depolarization with carbonyl cyanide m-chlorophenyl hydrazine (CCCP) or other pharmacological agents^20,27,28^. Endogenous Parkin is poorly expressed in several mammalian tissues and cell lines, and it is clear that mitophagy can occur independently of Parkin and PINK1^29,30^.

Here we report the identification of a previously undescribed role for the MEKK3-MEK5 signaling pathway as a regulator of basal mitophagy in cultured mammalian cells. MEKK3 and MEK5 are upstream activators of the extracellular signal-regulated kinase 5 (ERK5), which is the most recently discovered member of the mitogen-activated protein kinase (MAPK) family of proteins^31^. ERK5 is ubiquitously expressed in mammalian tissues and is activated by extracellular signals such as growth factors, several cellular stressors, and oxidative phosphorylation^31–33^. Mouse genetic studies have demonstrated that MEKK2/3-MEK5-ERK5 signaling is required for early embryogenesis, development of the vasculature and muscles, endothelial cell function, and cardio protection^31,32,34,35^. Alterations in the MEKK2/3-MEK5-ERK5 pathway have been associated with several human diseases and disorders including cancer, childhood obesity, scoliosis, and cerebral cavernous malformation^36–39^. Our studies indicate that MEKK3-MEK5-ERK5 signaling is critical for basal mitochondrial degradation in mammalian cells and plays a relevant role in erythrocyte maturation.

## Materials and methods

### Cell culture

Transformed MEFs, U2OS cells stably expressing mitochondrial-targeted mCherry, U2OS cells stably expressing GFP-LC3B, and HeLa YFP-Parkin cells were received from Michael White’s laboratory. The U2OS GFP-LC3B cells were originally a kind gift from Xiaodong Wang and the HeLa YFP-Parkin cells were originally a kind gift from Richard Youle. Mito-mKeima stably expressing U2OS cells were generated by transfection (Addgene #56018), selection with 1 mg/ml G418 for 2 weeks, and flow sorted for high expressers. Cells were grown in Dulbecco’s modified Eagle’s medium (DMEM) supplemented with 10% fetal bovine serum (FBS), l-Glutamine, Penicillin and Streptomycin, and Sodium Pyruvate, and at 37 °C and 5% CO_2_. Mito-mCherry, GFP-LC3B, and mito-mKeima stable cells were maintained in 1 mg/ml G418 DMEM media.

### CRISPR/Cas9-knockout cell lines

SQSTM1/p62- and MAPK7/ERK5-knockout U2OS cells were generated using CRISPR-Cas9 technology. Briefly, 400,000 U2OS cells were transiently transfected with precomplexed ribonuclear proteins consisting of 100 pmol of chemically modified single-guide RNA (sgSQSTM1 5′-gagggaaagggcuugcaccg-3′ or sgMAPK7 5′-agacggcgaggacggcucug-3′, Synthego), 35 pmol of Cas9 protein (St. Jude Protein Production Core), and 200 ng of pMaxGFP (Lonza) via nucleofection (Lonza, 4D-Nucleofector™ X-unit) using solution P3 and program CM-104 in a small (20 μl) cuvette according to the manufacturer’s recommended protocol.

Five days post nucleofection, cells were single-cell sorted by fluorescence-activated cell sorting to enrich for GFP+ (transfected) cells, clonally selected, and verified for the desired targeted modification via targeted deep sequencing using gene-specific primers with partial Illumina adapter overhangs (hSQSTM1.F 5′-acagccccacagtgacgacagaggg-3′ and hSQSTM1.R 5′-tgggggaggaattagcagagcggca-3′, or hMAPK7.F 5′-agctagtctgccacgaaccagccgc-3′ and hMAPK7.R 5′-cgggcggaggacaccactccatagg-3′, overhangs not shown). Next Generation Sequencing analysis of clones was performed using CRIS.py^40^. Genotypes of each clone are available upon request.

### Inhibitor treatments

Unless otherwise stated, cells were treated with 10 µM BIX02189 (Tocris 4842) and 10 µM XMD8–92 (Tocris 4132) for 16 h and with 50 nM Bafilomycin A1 (Sigma B1793) for 2 h.

### Immunoblotting

Western blottings were performed by lysing cells in 2× Laemmli loading buffer with 10 mM dithiothreitol, separated on polyacrylamide gels, transferred to polyvinylidene difluoride membranes, and probed with antibodies against p62/SQSTM1 (CS 51145), XPB (SC-293), TOMM40 (MBL 3740), B-actin (Sigma A1978), VDAC (CS 4867S), S6K (CS 9202S), ERK5 (CS 3372S), mtCOX2 (Ab797393), MEKK3 (SC-28769), MEK5 (Invitrogen PA5-15083), PGC1α (CS 2178S), and pERK5 (CS 3371S).

### Mitochondrial content

Relative mitochondrial content was assessed either qualitatively by western blotting for endogenous mitochondrial proteins and non-mitochondrial loading controls (described above) or via quantitative analysis of fluorescently labeled mitochondria via flow cytometry. Mitochondria were fluorescently labeled either by stable expression of mitochondrial-targeted mCherry (mito-mCherry) or staining with MitoTracker Green FM (Thermo Fisher, M7514). U2OS cells stably expressing mito-mCherry were plated in six-well plates for flow cytometry analysis or in Nunc Lab-Tek II chambered coverglass (Thermo, 155382) for microscopy imaging. Alternatively, cells were stained with 100 nM MitoTracker Green FM for 30 min then washed with phosphate-buffered saline (PBS) three times. Cells were then trypsinized and assayed as described in the Flow Cytometry section below. Mitochondrial content per cell was measured from 10,000 cells per condition by quantifying the fluorescence of either mito-mCherry or MitoTracker Green FM, and the median value for each condition was normalized to the median value from the appropriate control condition.

### Mitochondrial uncoupling

HeLa YFP-Parkin cells were treated with 10 nM CCCP overnight in addition to indicated treatments. Cells were then fixed and stained with 4′,6-diamidino-2-phenylindole (DAPI) and TOMM20 antibody.

### Confocal imaging

Regular confocal images were taken with Leica SP8 TCS equipped with White Light Laser, 405 nm diode laser using ×63/1.4NA/Oil/HC PL APO CS2/0.14 mm objective confocal microscope. Mito-mCherry images were created from Z-stacks taken at software calculated Nyquest pixel resolution. Higher resolution confocal images were taken using ×100/1.4NA objective with oversampled XY pixel size of 16 nm and Z-step size of 100 nm which were deconvolved using Huygens Professional 16.10 (Scientific Volume Imaging, The Netherlands).

### GFP-LC3B puncta and SQSTM1/p62 puncta were manually counted on a per cell basis

MAPK7/ERK5 wild-type and MAPK7/ERK5-knockout U2OS cells stably expressing mitochondrial-targeted mKeima were plated in Nunc Lab-Tek II chambered coverglass and imaged live on the Leica SP8 TCS confocal microscope. Data were analyzed by comparing 561 (acidic) signal to 458 (neutral) signal.

### Lysosomal acidification

Lysosomal content was determined by staining with LysoTracker Red DND-99 (L7528) at 100 nM for 30 min. Lysosomal acidification was determined by staining with LysoSensor Green DND-189 (L7528) at 1 mM for 10 min. Cells were then imaged using a BioTek Cytation 5 Cell Imaging Multi-Mode Reader. Cells were imaged using ×20 objective. Signal was analyzed via relative fluorescence using CellProfiler^41^.

### Flow cytometry

Cells were treated as indicated, trypsinized, spun down at 400 rpm, and resuspended in buffer made from 1× PBS, 2% serum, 1 mM EDTA, and 0.1% Sodium Azide. Cells were stained with DAPI. Fluorescence was then quantified on a BD Biosciences Fortessa by gating on single live cells and subsequently analyzed using FloJo v10.6.2.

### RNA sequencing

RNA was extracted from cultured cells using RNeasy Kit (QIAGEN #74104) following manufacturers’ protocols. RNA concentration was measured using a NanoDrop (Thermo Fisher Scientific, Waltham, MA) and the quality of RNA was determined with a bioanalyzer (Agilent Technologies, Santa Clara, CA). Libraries were prepared using the TruSeq Stranded Total RNA Library Prep Kit (Illumina, San Diego, CA) and subjected to 100 cycle paired-end sequencing on the Illumina HiSeq platform.

### Promega autophagy assay

HEK293 autophagy reporter cells (HiBiT-HaloTag-LC3, Promega #GA1040) were grown in in DMEM (Thermo Fisher #31053-028) supplemented with 10% FBS (Hyclone #SH30071), l-Glutamine (Thermo Fisher #25030-081), Penicillin and Streptomycin (Thermo Fisher #15140-122), Sodium Pyruvate (Thermo Fisher #11360-070), and 500 µg/mL G418 (Thermo Fisher #10131) at 37 °C and 5% CO_2_. Cells (2500) in 25 µl of G418-free media were plated into each well of Corning 8804BC 384-well plates and grown for 18 h. Cells were then treated with ten-point curves with threefold dilutions and a top concentration of 60 µM of Bafilomycin A1 (positive control for autophagy inhibition), Rapamycin (TOCRIS #1292; positive control for autophagy activation), BIX02188, BIX02189, and XMD8–92, or dimethylsulfoxide (DMSO) using a V&P Scientific S100 pin-tool before they were returned to 37 °C with 5% CO_2_ for 24 h. The assay plates were then equilibrated to room temperature for 15 min before the addition of 25 µl of Nano-Glo HiBiT Lytic Detection System (Promega #N3030). The plates were then shaken at 300 r.p.m. for 2 min on an orbital shaker, incubated for 30 min at room temperature, and the luminescence of each well measured using a Perkin Elmer Envision plate reader.

### Erythroblast maturation

Ter119-negative erythroid progenitors were isolated from embryonic day 13.5 or 14.5 Balb/c murine fetal livers as described^42^. The purity of the cells was confirmed by staining cells with PE-CD71 and allophycocyanin (APC)-Ter119 antibodies (all from BD Biosciences) by flow cytometry. Approximately 10% of total fetal livers were purified as Ter119 negative. These progenitors were set up on retronectin coated plates for differentiation and were followed over three days for differentiation status and mitochondrial content. The data were acquired on a BD FACSCalibur and analyzed by FlowJo 8.8.4. To measure mitochondrial content, cells were stained with 25 nM MitoTracker Deep Red FM (Molecular Probes, Invitrogen) for 30 min at 37 °C.

For inhibitor treatments starting on Day 0, Ter119-negative progenitors were set up for differentiation directly after purification in erythropoietin (Epo) media in the presence of inhibitor on retronectin coated plates. For inhibitor treatments starting on Day 2 and Day 3 Ter119-negative progenitors, 20 h after culturing in Epo medium or 1 day after removal from EPO medium, were set up for differentiation separately, and inhibitors were added when indicated. Erythroid differentiation was monitored on days 1, 2, and 3 with PE-CD71 (BD Biosciences) and APC-Ter119 (eBioscience) by flow cytometry. 7-AAD (BD Biosciences) staining was used to exclude dead cells. Cells were treated with DMSO, 10 µM BIX02188, or 10 µm XMD8–92.

## Results

### SQSTM1/p62 constitutively delivers mitochondria to lysosomes for degradation in the absence of exogenous damage

**Fig. 1:**
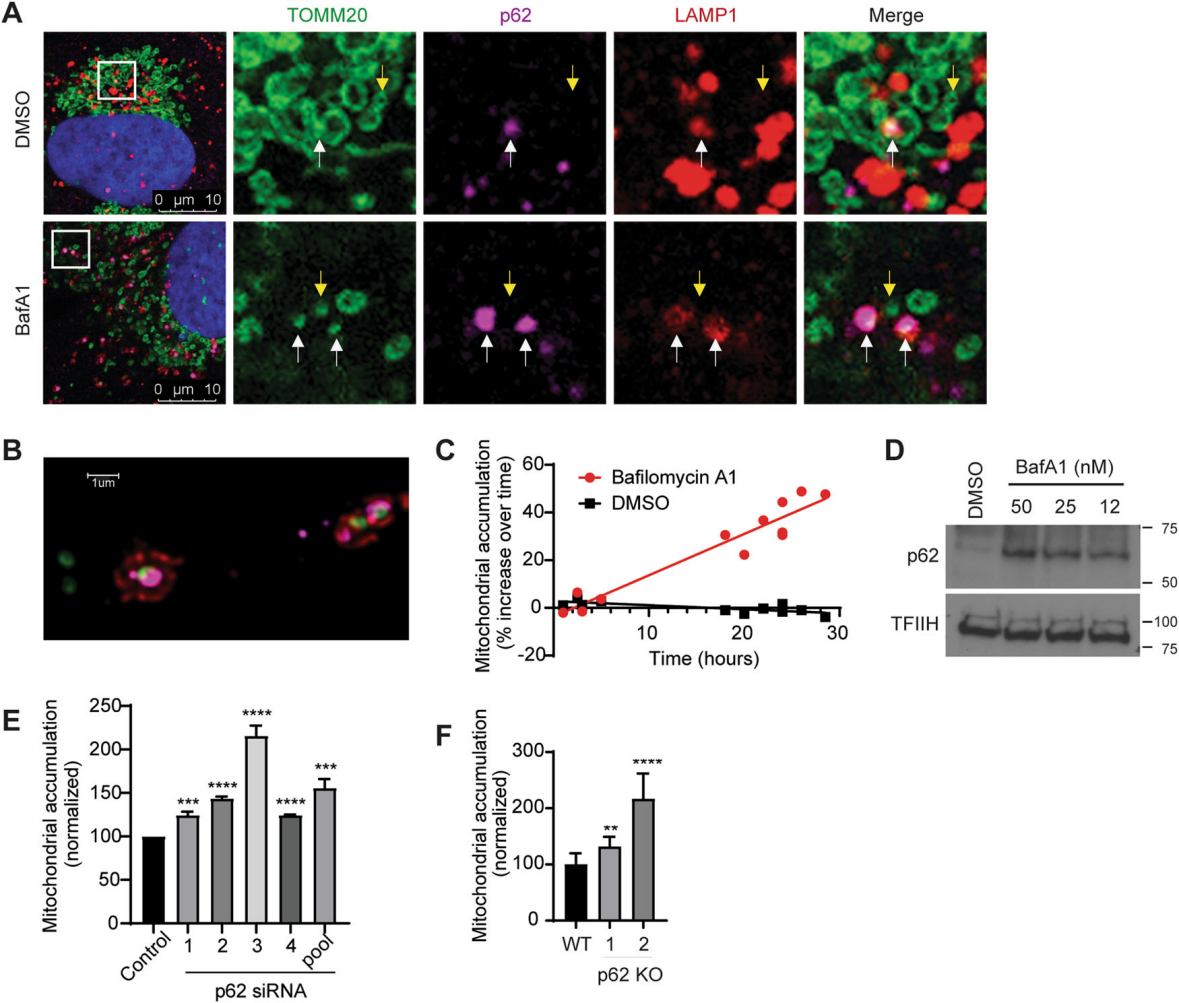
SQSTM1/p62 constitutively delivers mitochondria to lysosomes for degradation in the absence of exogenous damage. **a** U2OS cells were treated with vehicle (DMSO) or with 50 nM Bafilomycin A1 for 2 h to block degradation of lysosomal contents and analyzed by fluorescence confocal microscopy. Representative images are shown. TOMM20 (Green), p62 (purple), LAMP1 (red), Dapi (blue). White arrows show instances of mitochondria tagged by p62 and surrounded by lysosomes. Yellow arrows designate mitochondria that are not being degraded. **b** Representative high-resolution confocal image of cells treated with bafilomycin A1 as in **a**. Tomm20 (green), p62 (purple), LAMP1 (red). **c** U2OS mito-mCherry cells were treated with 50 nM Bafilomycin A1 or vehicle (DMSO) for the indicated time. Mitochondrial accumulation was detected by flow cytometry and displayed as percent increase over time. **d** Protein levels of p62 from U2OS cells that were treated with the indicated concentrations of Bafilomycin A1 overnight were detected by western blotting analysis. TFIIH was used as a loading control. **e** U2OS mito-mCherry cells were transfected with the indicated siRNA oligos or siControl (the p62 Si pool contains oligos 1–4). Seventy-two hours later, mitochondrial accumulation was measured by flow cytometry. Mean ± SE of *n* = 3 independent experiments is shown; ***p < 0.001; ****p < 0.0001. **f** p62-knockout U2OS cells were stained with 100 nM MitoTracker Green FM for 30 min, and mitochondrial accumulation was measured by flow cytometry. Mean ± SE of *n* = 7 independent experiments is shown; **p < 0.01; ****p < 0.0001.

The primary question we set out to address is what controls basal mitochondrial degradation in the absence of exogenous damage. This process is known to be mechanistically distinct from damage-induced mitophagy, which requires the kinase PINK1, the E3 ligase Parkin, and the selective autophagy receptors NDP52 and Optineurin^28,30^. We first investigated whether an alternative selective autophagy receptor, SQSTM1/p62, supports mitochondrial degradation in the absence of exogenous damage. We selected p62 as an initial candidate because it cannot support damage-induced, Parkin-dependent mitophagy but has been reported to be important for mitochondrial degradation in other contexts^17,28,43–45^. We treated U2OS osteosarcoma cells with Bafilomycin A1 for 2 h to inhibit degradation of all cargo delivered to the lysosome and used immunofluorescent microscopy to determine whether such cargo included mitochondria and p62. Our experiments revealed that a minority of the mitochondrial population (marked by TOMM20) were delivered to LAMP1 (lysosomal associated membrane protein 1)-positive lysosomes in the absence of any mitophagy-inducing treatment, and all LAMP1-encapsulated mitochondria were co-labeled with p62 (Fig. 1a, b). In contrast, the vast majority of LAMP1-negative mitochondria were negative for p62 (Fig. 1a, b). In the absence of Bafilomycin treatment we observed rare instances of p62-positive mitochondria co-localized with LAMP1 (Fig. 1a).

To determine whether lysosomal activity detectably limited mitochondrial accumulation under basal conditions, we stably expressed a construct encoding the CoxVIII leader sequence fused to mCherry in U2OS cells (U2OS mito-mCherry cells) and measured mCherry fluorescent intensity per cell by flow cytometry as a marker of mitochondrial abundance. Short durations of lysosomal inhibition (<5 h) did not detectably increase bulk mitochondrial content per cell, whereas longer durations of lysosomal inhibition caused a steady increase of mitochondrial content over time (Fig. 1c). Together these results indicate that steady-state mitochondrial abundance is limited by ongoing delivery of a small subset of p62-labeled mitochondria to lysosomes for degradation. Treatment with Bafilomycin A1 (BafA1) increased total protein levels of p62 in a dose-dependent manner, confirming that p62 is continually degraded by the lysosome under basal conditions (Fig. 1d). Together, these results suggest that p62 labels mitochondria destined for delivery to lysosomes in the absence of exogenous damage or ectopic Parkin overexpression.

To test whether p62 is required for basal mitochondrial degradation, we depleted p62 by RNA interference (RNAi) or CRISPR/Cas9-mediated gene editing and asked whether total mitochondrial content per cell increased as a result. Multiple independent siRNA oligos targeting p62 caused an increase in mitochondrial accumulation in U2OS mito-mCherry cells (Fig. 1e and Supplementary Fig. 1A). Similarly, p62-knockout U2OS cells exhibited increased mitochondrial accumulation relative to parental U2OS cells as measured by MitoTracker Green FM staining (Fig. 1f and Supplementary Fig. 1B). Taken together, our data indicate p62 is important for basal delivery of mitochondria to lysosomes for degradation in the absence of exogenous damage.

### Nomination of putative mitophagy regulatory pathways

**Fig. 2:**
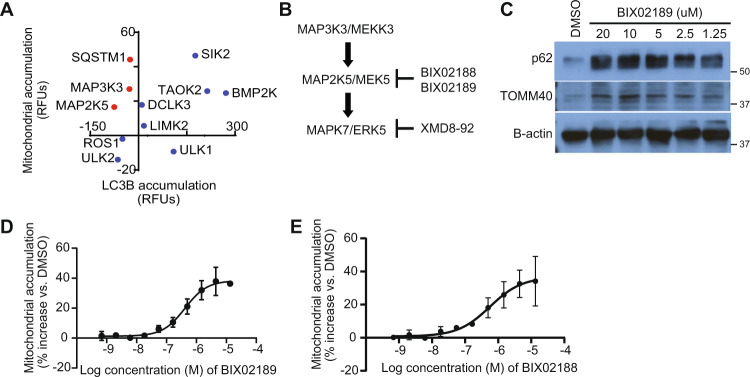
Nomination of putative mitophagy regulatory pathways. **a** Candidate mitophagy-regulating genes identified by FuSiOn were screened by siRNA-mediated depletion in U2OS mito-mCherry cells and U2OS GFP-LC3B cells. Red dots denote selectivity of candidate gene for mitochondrial degradation. **b** Model of the MAP3K3 kinase cascade including inhibitors. **c** Protein levels of p62 and mitochondrial marker TOMM40 from mouse embryonic fibroblasts treated with vehicle (DMSO) or the indicated concentrations of the BIX02189 overnight. **d** Mitochondrial accumulation in U2OS mito-mCherry cells treated overnight with vehicle (DMSO) or BIX02189 was measured by flow cytometry. Mean ± SD of *n* = 2 independent experiments is shown. **e** Mitochondrial accumulation in U2OS mito-mCherry cells treated overnight with vehicle (DMSO) or BIX02188 was measured by flow cytometry. Mean ± SD of *n* = 3 independent experiments is shown.

To identify novel mitophagy regulatory pathways, we utilized Functional Signature Ontology (FuSiOn), a method for unbiased identification of functionally related proteins, to nominate protein kinases from the human genome that function similarly to p62 under basal conditions^46^. FuSiOn identified ten kinases (BMP2K, DCLK3, LIMK2, MAP2K5/MEK5, MAP3K3/MEKK3, ROS1, SIK2, TAOK2, ULK1, and ULK2) whose depletion mimicked the phenotypic effect of p62 depletion in HCT116 colon cancer cells^46^. To determine whether any of these ten candidates promotes basal mitophagy, we depleted each kinase individually in U2OS mito-mCherry cells and measured the resulting change in average mitochondrial content per cell. Depletion of seven of the ten kinases caused an increase in mitochondrial content similar to that caused by depletion of p62 (Fig. 2a). Next, we depleted each kinase in U2OS GFP-LC3B cells and measured accumulation of GFP-LC3B as a marker of nonselective autophagy inhibition. Two candidates (*MAP3K3* and *MAP2K5*) phenocopied p62’s selectivity for mitochondria, demonstrated by the relatively consistent LC3B levels in addition to the increase in mitochondrial content (Fig. 2a). *MAP3K3* encodes MEKK3, which activates the MEK5-ERK5 kinase cascade. *MAP2K5* encodes the MEKK3 substrate MEK5 (Fig. 2b). We confirmed on-target efficacy of the siRNA pools directed against MAP3K3/MEKK3 and MAP2K5/MEK5 (Supplementary Fig. 1C). Maximum intensity projection images of U2OS mito-mCherry cells confirmed that the mito-mCherry signal remained localized to mitochondrial network after depletion of MEKK3 or MEK5 and demonstrated the accumulation of mitochondrial network in individual cells, supporting the flow cytometric data (Supplementary Fig. 1D). Next, we tested whether pharmacological inhibition of MEK5 kinase activity could alter mitochondrial abundance using two small molecule inhibitors of MEK5 (BIX02188 and BIX02189)^47^. Both BIX02188 and BIX02189 inhibited MEK5 activity and increased mitochondrial content in a dose-dependent manner in varied mammalian cells (Fig. 2c-e and Supplementary Fig. 1E). Given the structural similarity between the two inhibitors, we measured the off-target activities of BIX02188 and BIX02189 by Kinome Profiling and determined that the off-target activities do not overlap (Supplementary Table 1). This increases the likelihood that the observed increase in mitochondrial content is due to inhibition of the intended target, MEK5. Together, these results indicate that MEKK3-MEK5 signaling restrains mitochondrial accumulation.

### The MEKK3-MEK5-ERK5 kinase cascade prevents accumulation of excess mitochondria

**Fig. 3:**
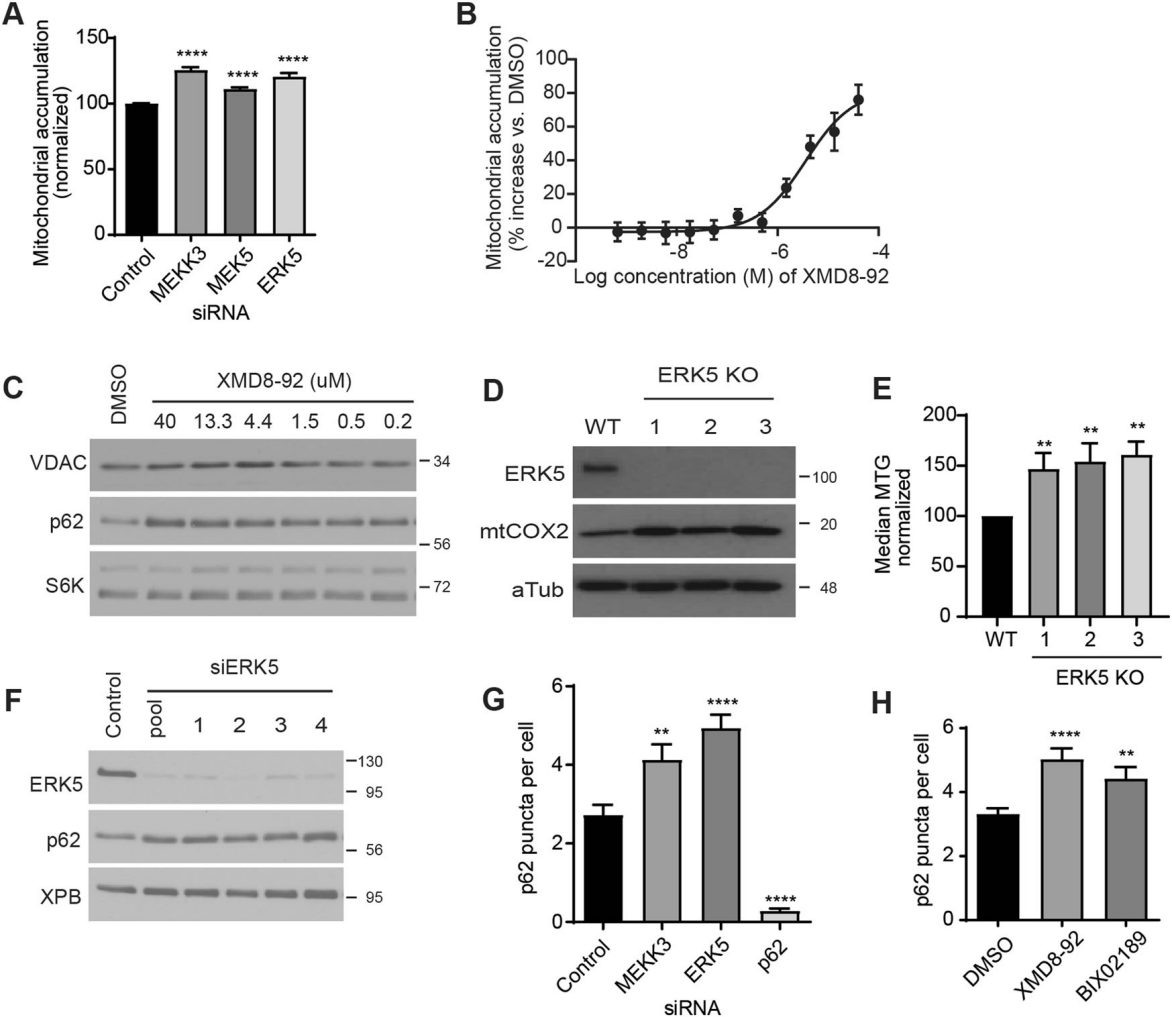
The MEKK3-MEK5-ERK5 kinase cascade prevents accumulation of excess mitochondria. **a** U2OS mito-mCherry cells were transfected with the indicated siRNA oligos. Seventy-two hours later, mitochondrial accumulation was analyzed by flow cytometry. Mean ± SD of *n* = 3 independent experiments is shown. **b**, **c** Mitochondrial accumulation in U2OS mito-mCherry cells treated overnight with vehicle (DMSO) or XMD8–92 was measured by flow cytometry or western blot, respectively. Mean ± SD of *n* = 2 independent experiments is shown **b**. **d**, **e** Mitochondrial levels of three independent MAPK7/ERK5-knockout U2OS clones were assessed by western blotting analysis of mitochondrial marker mtCOX2 (**d**) or by MitoTracker Green FM staining and flow cytometry (**e**). **f** Protein levels of p62 from U2OS cells that were treated with ERK5 siRNA for 72 h were detected by western blotting analysis. XPB was used as a loading control. **g**, **h** U2OS cells were either transfected with the indicated siRNAs for 72 h (**g**) or treated with the indicated drug at 10 µM overnight (**h**). Cells were fixed and stained with p62 antibody and then imaged. See Supplementary Fig. 1f, g for representative images.

We proceeded to assay whether the canonical downstream component of this kinase cascade, ERK5 (encoded by the *MAPK7* gene), also plays a role in restraining mitochondrial accumulation. RNAi-mediated depletion of ERK5 increased the average mitochondrial content per cell, indicating that ERK5 (like p62, MEKK3, and MEK5) prevents excess accumulation of mitochondria under basal conditions (Fig. 3a and Supplementary Fig. 1D). A small molecule inhibitor of ERK5, XMD8–92^48^, also increased mitochondrial content in a dose-dependent manner (Fig. 3b, c). Thus, we generated MAPK7/ERK5-knockout U2OS cells using CRISPR/Cas9 technology and analyzed mitochondrial content via western blotting and MitoTracker Green FM staining. ERK5-knockout cells exhibited increased mitochondrial accumulation relative to the parental controls (Fig. 3d, e). These data indicate that the canonical MEKK3-MEK5-ERK5 kinase cascade restrains mitochondrial accumulation under basal conditions.

We asked whether the MEKK3-MEK5-ERK5 kinase cascade promotes mitochondrial degradation through regulation of p62 protein levels. MEKK3, MEK5, and p62 all contain PB1 domains, which mediate protein–protein dimerization^49^. We hypothesized that MEKK3-MEK5-ERK5 pathway inhibition might decrease p62 protein stability, or alternatively, might reduce p62 expression given that ERK5 is known to translocate to the nucleus and regulate gene transcription when activated^31,50^. In either of these cases, a decrease in p62 levels upon MEKK3-MEK5-ERK5 pathway inhibition could explain why mitochondria accumulate under those conditions.

However, genetic or pharmacological inactivation of the MEKK3-MEK5-ERK5 kinase cascade induced p62 protein accumulation rather than decrease in abundance, indicating that MEKK3-MEK5-ERK5 signaling is not required for p62 expression or stability (Figs. 2c and 3c, f). Using immunofluorescent microscopy, we found that genetic or pharmacological inactivation of the MEKK3-MEK5-ERK5 kinase cascade caused an increase in the average number of cytoplasmic p62 puncta per cell (Fig. 3g, h and Supplementary Fig. 1F, G). The punctate accumulation of the selective autophagy adaptor protein p62 upon inhibition of the MEKK3-MEK5-ERK5 pathway is consistent with the interpretation that this pathway promotes one or more forms of selective autophagy under basal conditions.

### The MEKK3-MEK5-ERK5 pathway is required for lysosomal degradation of mitochondria

**Fig. 4:**
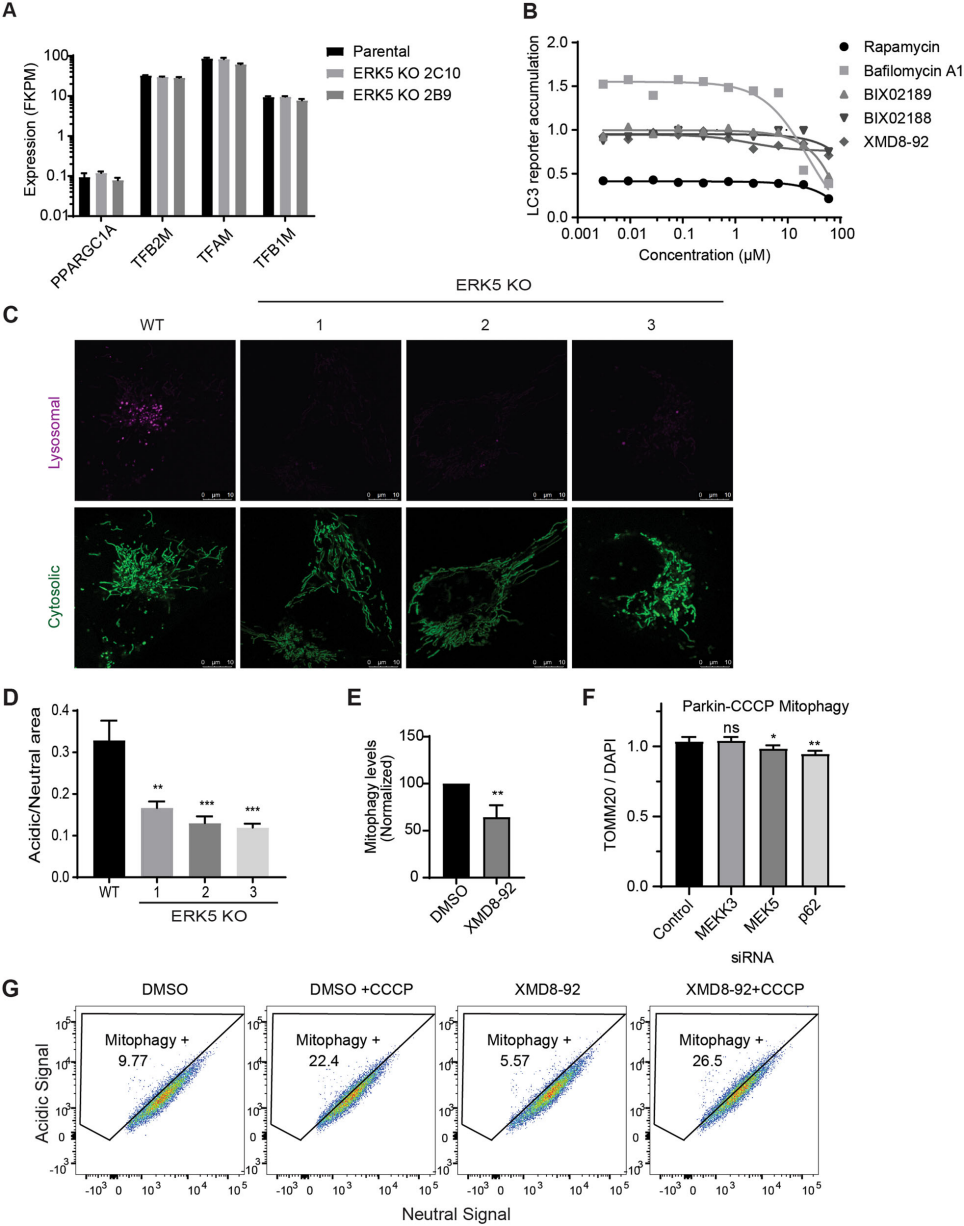
The MEKK3-MEK5-ERK5 pathway is required for lysosomal degradation of mitochondria. **a** RNA-seq was performed on parental U2OS cells and two MAPK7/ERK5-knockout U2OS clones. Expression of several mRNAs encoding mitochondrial biogenesis genes is shown. **b** HEK293 cells stably expressing the HiBiT-LC3 bulk-autophagy reporter were treated with vehicle (DMSO) or the indicated compounds for 24 h in duplicate. Accumulation of the reporter was measured using the Promega Autophagy Assay system and normalized to vehicle-treated cells. **c**, **d** Representative images (**c**) and quantification (**d**) of active mitophagy events in parental (WT) and ERK5-knockout U2OS cells expressing mitochondrial-targeted mKeima. **e** Mitophagy levels in parental U2OS cells expressing mitochondrial-targeted mKeima were assessed by flow cytometry and quantified. Cells were treated overnight with either DMSO or 10 µM XMD8–92. Mean ± SD of *n* = 3 independent experiments is shown. **f** HeLa YFP-Parkin stable cells were transfected with the indicated siRNA for 72 h and treated with 10 µM CCCP overnight. Cells were fixed, stained with anti-Tom20 and DAPI, and imaged. Mitochondrial content was quantified and normalized to DAPI. **g** WT U2OS cells expressing mitochondrial-targeted mKeima were treated overnight with vehicle control, 10 µM CCCP, 10 µM XMD8–92, or both. Mitophagy levels were analyzed via flow cytometry.

To determine the underlying cause of increased mitochondrial content observed upon inhibition of MEKK3-MEK5-ERK5 signaling, we considered and tested three distinct possibilities: (1) induction of mitochondrial biogenesis; (2) nonselective inhibition of the autophagy-lysosome pathway; and (3) selective inhibition of mitochondrial degradation. PGC1α is a critical regulator of mitochondrial biogenesis^51^, and increased protein levels and activation of PGC1α leads to an increase in transcription factors TFAM, TFB1M, and TFB2M^52^. Therefore, if inhibition of MEKK3-MEK5-ERK5 signaling increases mitochondrial abundance by inducing mitochondrial biogenesis, we should detect increased expression of PGC1α, TFAM, TFB1M, and TFB2M upon inhibition of the pathway. RNA sequencing confirmed that there was no increase in the mRNA levels of these major mitochondrial biogenesis-related transcription factors upon knockout of ERK5 (Fig. 4a and Supplementary Table 2). We also verified that the protein levels of the master mitochondrial biogenesis factor PGC1α did not increase upon inhibition of the MEKK3-MEK5-ERK5 pathway. In fact, PGC1α levels decreased upon pharmacological inhibition of MEK5 or ERK5 (Supplementary Fig. 2A), consistent with existing literature reporting a positive role for ERK5 signaling in promoting mitochondrial biogenesis^35^. Therefore, MEKK3-MEK5-ERK5 signaling does not restrain mitochondrial accumulation by limiting mitochondrial biogenesis.

In addition, MEK5/ERK5 signaling is not required for nonselective autophagy or for general lysosomal function. We performed a Promega Autophagy Assay, in which we treated HEK293 cells expressing the HiBiT-LC3 autophagy reporter with a range of concentrations of DMSO, Bafilomycin A1, Rapamycin, BIX02188, BIX02189, and XMD8–92 for 24 h. As expected, Bafilomycin A1 effectively inhibited nonselective autophagy and Rapamycin effectively induced autophagy across wide dose ranges, as evidenced by increased (Bafilomycin) or decreased (Rapamycin) accumulation of the HiBiT-LC3 reporter (Fig. 4b). In contrast, BIX02188, BIX02189, and XMD8–92 did not inhibit nonselective autophagy even at concentrations as high as 60 µM (Fig. 4b). Furthermore, we determined that inhibition of the MEK5-ERK5 pathway does not cause lysosomal deacidification or alter lysosomal content (Supplementary Fig. 2B, C). We also monitored autophagosome formation and autophagic flux using U2OS GFP-LC3B cells and observed no disruptions in either process upon treatment with XMD8–92 (Supplementary Fig. 2D, E).

The combined findings indicate the MEKK3-MEK5-ERK5 kinase cascade restrains mitochondrial accumulation independent of mitochondrial biogenesis, driving bulk autophagy, or supporting general lysosome function. Next, we questioned whether the MEKK3-MEK5-ERK5 kinase cascade is required for selective degradation of mitochondria. Therefore, we developed WT and ERK5 KO U2OS cell lines expressing mitochondrial-targeted monomeric Keima, a pH-dependent fluorescent protein^53,54^ to measure the delivery of mitochondria to lysosomes. When targeted to the mitochondria, the pH-dependent fluorescence allows us to distinguish between mitochondria in the cytoplasm (458 nm) and mitochondria that are being degraded in the highly acidic lysosome (561 nm)^55^. We found that all three independent ERK5-knockout clones had significantly less mitochondria in the lysosomes compared to parental cells (Fig. 4c, d). Furthermore, in parental U2OS mito-mKeima cells, pharmacological inhibition of ERK5 using XMD8–92 decreased the amount of basal mitophagy compared to DMSO control (Fig. 4e). However, inhibition of the MEKK3-MEK5-ERK5 pathway by genetic or pharmacological tools did not mitigate CCCP-induced mitophagy either in the absence or presence of ectopic Parkin overexpression (Fig. 4f, g and Supplementary Fig. 2F-I). The combined data indicate the MEKK3-MEK5-ERK5 pathway specifically promotes lysosome-mediated mitochondrial degradation under basal conditions and is not required for nonspecific bulk autophagy, general lysosome function, or damage-induced mitophagy nor for restraint of mitochondrial biogenesis.

### MEKK3-MEK5-ERK5 pathway is required for differentiation and mitophagy in erythroid progenitors

**Fig. 5:**
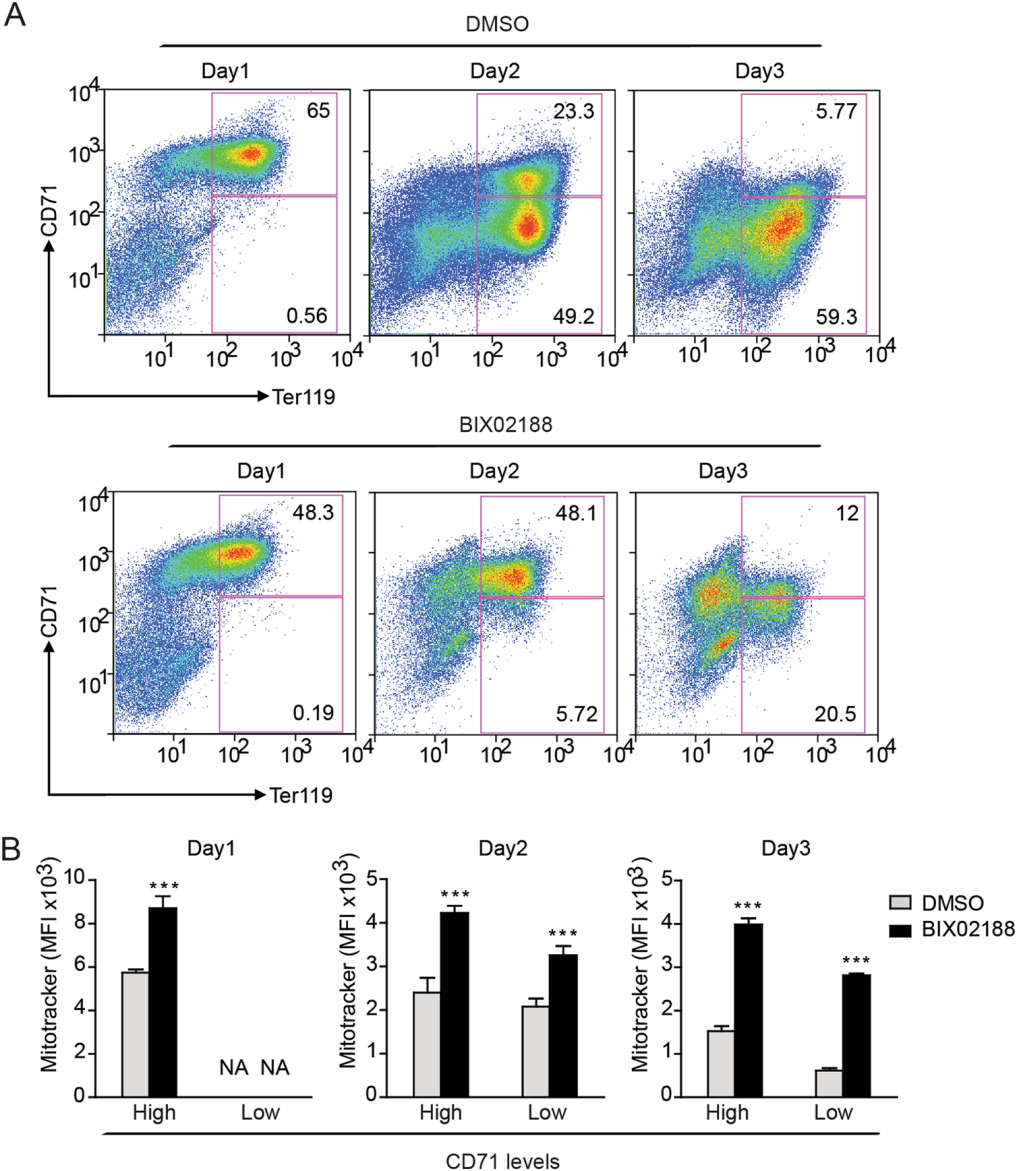
MEK5 is required for differentiation and mitophagy in erythroid progenitors. **a** Murine erythroid progenitors were treated with the BIX02188 (30 μM) or vehicle control (DMSO) prior to initiation of differentiation. Levels of CD71 and Ter119 were measured by flow cytometry. Representative profiles are shown. **b** Mitochondrial mass was quantified in Ter119^+^CD71^high^ and Ter119^+^CD71^low^ erythroblasts using median fluorescence intensity (MFI) of MitoTracker Deep Red FM. The data represent three replicates. ***P < 0.001.

**Fig. 6:**
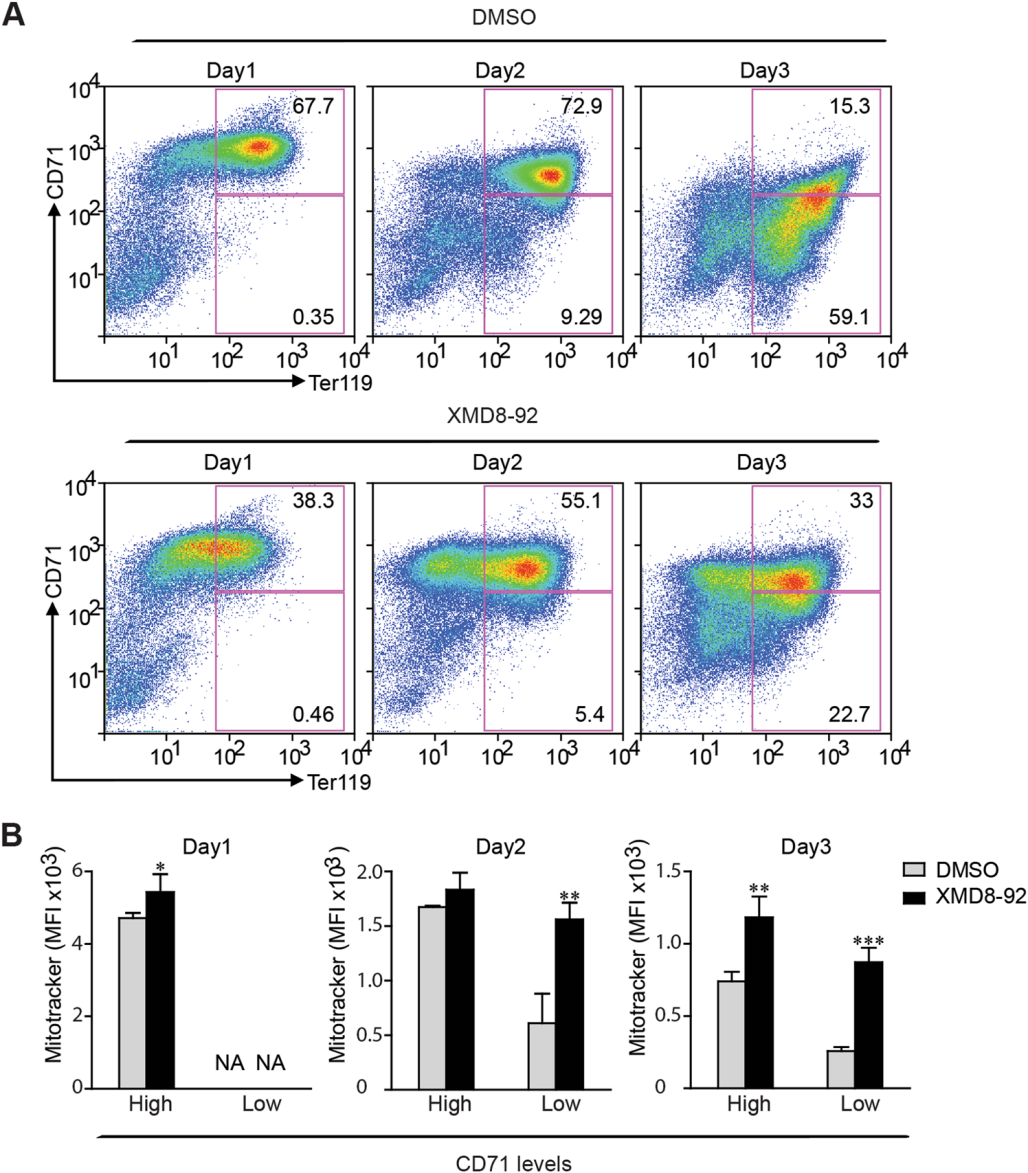
ERK5 is required for differentiation and mitophagy in erythroid progenitors. **a** Murine erythroid progenitors were treated with XMD8–92 (5 μM) or vehicle control (DMSO) prior to initiation of differentiation. Levels of CD71 and Ter119 were measured by flow cytometry. Representative profiles are shown. **b** Mitochondrial mass was quantified in Ter119^+^CD71^high^ and Ter119^+^CD71^low^ erythroblasts using median fluorescence intensity (MFI) of MitoTracker Deep Red FM. The data represent three replicates. *P < 0.05; **P < 0.01; ***P < 0.001.

Previous studies suggest that ERK5-deficient cells develop altered nucleotide metabolism, impairing erythropoiesis in mice^56^. Erythropoiesis involves an ordered acquisition and loss of key differentiation markers^57^. As erythroid progenitors must eliminate their mitochondria to fully mature, we evaluated whether ERK5-regulated mitochondrial degradation participates in erythrocyte maturation^58^. To investigate the possible role of the MEKK3-MEK5-ERK5 pathway in erythrocyte maturation, Ter119-negative erythroid progenitors were isolated from murine fetal livers and induced to differentiate by addition of Epo ex vivo. We followed the expression of two specific differentiation markers: CD71, the transferrin receptor, whose expression decreases as erythroblasts mature, and Ter119, an antigen expressed on cell surfaces of more mature erythroblasts^57^. BIX02188 or vehicle control (DMSO) were present throughout the ex vivo differentiation process. Pharmacological inhibition of MEK5 impaired erythroid differentiation as evidenced by reduced percentages of cells reaching the most advanced Ter119^+^CD71^low^ stage of differentiation (Fig. 5a). Notably, MEK5 inhibition significantly increased basal mitochondrial content at all stages of differentiation (Fig. 5b). As differentiation progressed, both BIX02188-treated and DMSO-treated cells lost mitochondrial content over time; however, mitochondrial content was always significantly higher in BIX02188-treated cells relative to DMSO-treated cells at each stage (Fig. 5b). Similar results were observed when ERK5 was inhibited using XMD8–92 (Fig. 6a, b). Erythroid differentiation was similarly inhibited by genetic depletion of MEK5 using short hairpin RNA (Supplementary Fig. 3). Together, these data indicate that MEK5-ERK5 signaling is required for proper erythroid differentiation and promotes clearance of mitochondria.

## Discussion

Currently, there is substantial interest in the role autophagy plays in maintaining normal physiology and how dysfunctional mitophagy can lead to or enhance diseases. Our discovery of the MEKK3-MEK5-ERK5 pathway as a novel mitophagy regulator could facilitate a deeper understanding of how different mechanisms act to maintain homeostasis and prevent disease in different tissues. Using genetic and pharmacological inhibition of the MEKK3-MEK5-ERK5 pathway, we observed mitochondrial content increase in human cancer cells, mouse embryonic fibroblasts, and primary erythroid progenitor cells isolated from the murine fetal liver. This suggests that MEKK3-MEK5-ERK5 restrains mitochondrial accumulation in diverse cells and tissues. We also observed an increase in the levels of mitophagy receptor protein p62 after pharmacological or genetic inhibition of MEKK3-MEK5-ERK5 signaling. We confirmed that inhibition of pathway activity results in decreased lysosome-dependent degradation of mitochondria using mitochondrial-targeted mKeima, without affecting lysosome acidification or nonselective bulk-autophagy levels in the cell. Taken together, we found that the MEKK3-MEK5-ERK5 pathway promotes mitophagy without the addition of exogenous mitochondrial damage, which could have great implication on studying mitophagy in the context of development and disease.

In addition, our findings provide evidence for the importance of p62 in mitophagy under basal conditions. We have found that SQSTM1/p62 routinely delivers mitochondria to lysosomes without the addition of exogenous damage. We have found that inhibition of autophagy non-specifically with Bafilomycin A1 increases total mitochondrial load in cells, indicating that mitophagy is an ongoing maintenance process. Whether MEKK3-MEK5-ERK5 signaling controls mitochondrial degradation by directly or indirectly regulating p62 remains to be determined, although we have ruled out the possibility that MEKK3-MEK5-ERK5 signaling is required for p62 expression or stability. Direct interactions between the PB1 domain of p62 and the PB1 domains of MEKK3 and MEK5 have been reported, providing a potential means for direct regulation of p62 by the MEKK3-MEK5-ERK5 kinase cascade^49,59^.

We found that basal mitochondrial degradation, which requires p62 and MEKK3-MEK5-ERK5 signaling, is quite distinct from damage-induced mitophagy, which does not require p62 or MEKK3-MEK5-ERK5. We sought to investigate whether developmentally programmed mitochondrial degradation in the erythrocyte lineage required MEK5-ERK5 signaling as a well-established model of mitochondrial clearance in the absence of exogenous damage. Although impaired maturation of erythrocytes upon MEK5 or ERK5 inhibition precluded full assessment of the impact on mitochondrial turnover, the mitochondrial content was increased at each stage of erythroid maturation in MEK5-ERK5 inhibited cells. Taken together with the lack of a requirement for MEKK3-MEK5-ERK5 signaling in CCCP-induced mitophagy, it appears that the MEKK3-MEK5-ERK5 pathway may be solely designated for basal mitochondrial clearance. It is also possible that MEKK3-MEK5-ERK5 signaling may be required for induction of mitochondrial degradation by other stimuli, such as statin treatment, hypoxia, or forced metabolic switch from glycolytic metabolism to oxidative phosphorylation^60–63^. These questions remain to be addressed in future work.

Although MEKK3-MEK5-ERK5 signaling has been implicated in many aspects of mammalian development and disease, it is also the most recently discovered MAP kinase pathway, and much work needs to be done to fully understand how the MEKK3-MEK5-ERK5 kinase cascade is acting. It will be interesting to determine whether any of the previously identified roles for the ERK5 pathway in development, physiology, or disease represent an underlying role for mitochondrial degradation. For example, ERK5 signaling is well established to play an anti-apoptotic role, and mitochondria harbor several pro-apoptotic molecules including cytochrome *c*, Smac/DIABLO, and AIF^31,64,65^. It is possible that active ERK5 signaling antagonizes autophagy in part through elimination of pro-apoptotic molecules via delivery of mitochondria to lysosomes for degradation. These and other interesting hypotheses arising from our current work remain to be tested in future studies.

## Supplementary information

Supplemental Figure Legends

Supplementary Figure S1

Supplementary Figure S2

Supplementary Figure S3

Supplementary Table S1

Supplementary Table S2
