# Supplementary material for: MEKK3-MEK5-ERK5 signaling promotes mitochondrial degradation
Source: Cell Death Discov. 2020 Oct 20;6:107. doi: 10.1038/s41420-020-00342-7 (PMC7576125; doi:10.1038/s41420-020-00342-7)
Supplement: Supplementary file 1 — Supplemental Figure Legends [file 41420_2020_342_MOESM1_ESM.docx]

# Supplemental Figure Legends

## Supplementary Figure 1: The MEKK3-MEK5-ERK5 pathway prevents accumulation of excess mitochondria.

**a** p62 protein levels in U2OS cells 72 hours after transfection with the scrambled and p62 siRNAs. **b** Parental and p62 knockout U2OS cell lysates were collected and analyzed by western blot using the indicated antibodies. **c** Protein depletion by siRNA was analyzed by western blot in U2OS cells 72 hours after transfection. **d** Maximum intensity projections showing representative mitochondrial content in U2OS mito-mCherry cells 72 hours after siRNA transfection. **e** Phosphorylated ERK5 proteins levels in mouse embryonic fibroblasts were treated with vehicle (DMSO) or BIX02189 overnight. **f, g** U2OS cells were either transfected with the indicated siRNAs for 72 hours or treated with the indicated drug at 10 µM overnight, respectively. Cells were fixed and stained with p62 antibody, and then imaged.

## Supplementary Figure 2: The MEKK3-MEK5-ERK5 pathway does not inhibit mitochondrial biogenesis or drive bulk autophagy.

**a** PGC1α protein levels were analyzed by western blot from U2OS cells treated overnight. **b, c** Lysosomal content and acidification was measured by staining with LysoTracker Red and LysoSensor Green respectively. Cells were then imaged and relative fluorescence was quantified. **d, e** U2OS GFP-LC3B cells were imaged to assess the subcellular localization of GFP-LC3B. Cells were treated with DMSO, XMD8-92 (10 μM), Bafilomycin A1 (50 nM), or XMD8-92 + Bafilomycin A1 for two hours. Quantification of GFP-LC3B-positive puncta per cell from 19-21 cells per condition is shown. **f, g** Mitochondrial content in HeLa YFP-Parkin stable cells treated with either vehicle control (DMSO) or 10 µM CCCP overnight. Individual cells were categorized as exhibiting either mitochondrial retention, partial loss, or complete loss using TOMM20 fluorescence signal. **h, i** Mitochondrial content in HeLa YFP-Parkin stable cells co-treated with 10 µM CCCP and either vehicle control, BIX02189, or XMD8-92 overnight. The fraction of individual cells exhibiting complete mitochondrial (marked by TOMM20) loss under each condition is quantified.

## Supplementary Figure 3: Inhibition of MEK5 impairs erythroid differentiation

Erythroid progenitors were transduced with shRNA for MEK5 or control shRNA prior to erythroid differentiation. Stages of differentiation were identified by CD71 and Ter119 staining via flow cytometry. Transduced cells, identified by GFP expression, were gated for analyses. Representative flow cytometric profiles are shown.
