# Supplementary figures and images for: MEKK3-MEK5-ERK5 signaling promotes mitochondrial degradation
Source: Cell Death Discov. 2020 Oct 20;6:107. doi: 10.1038/s41420-020-00342-7 (PMC7576125; doi:10.1038/s41420-020-00342-7)

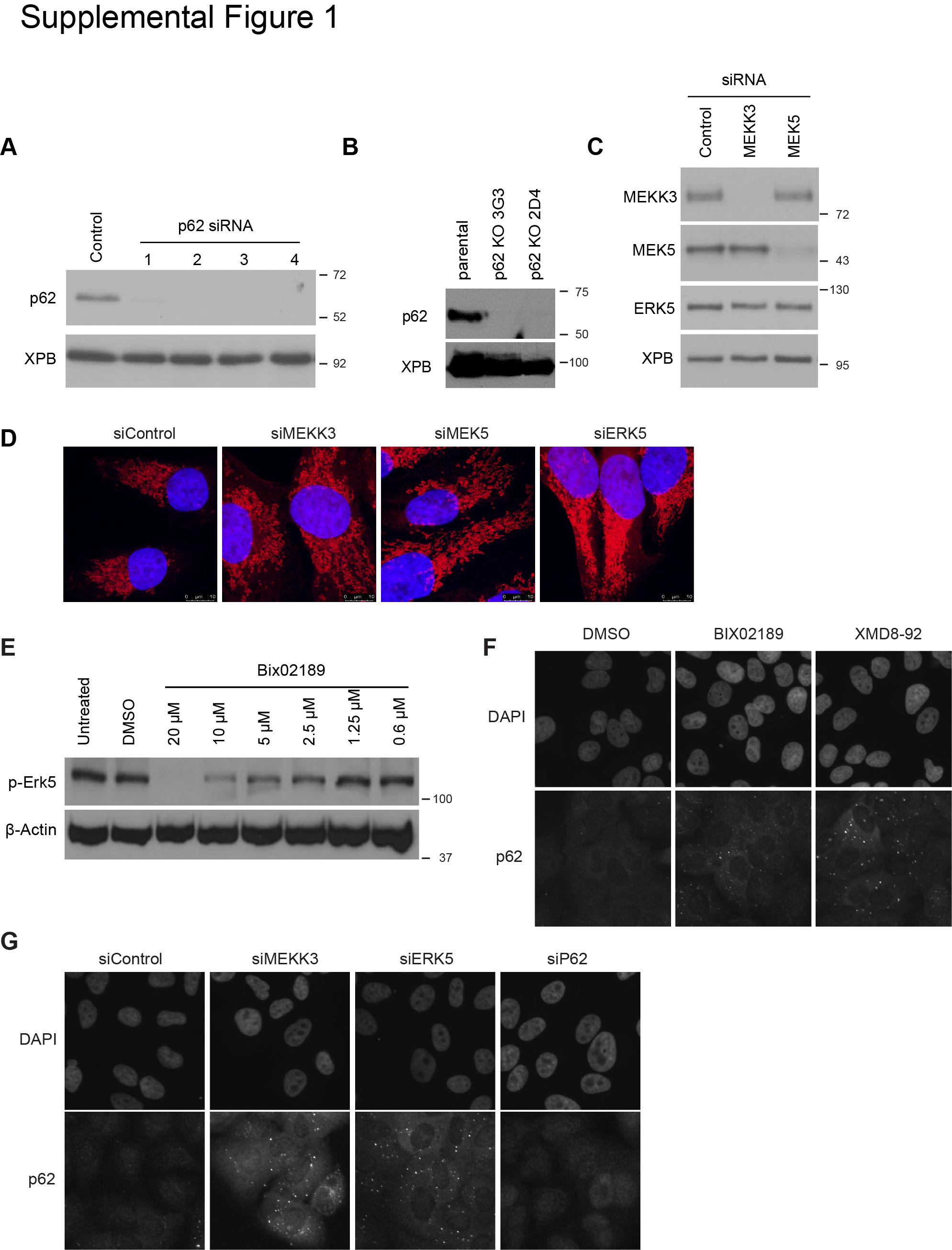

Supplement: Supplementary file 2 — Supplementary Figure S1 [file 41420_2020_342_MOESM2_ESM.tif]

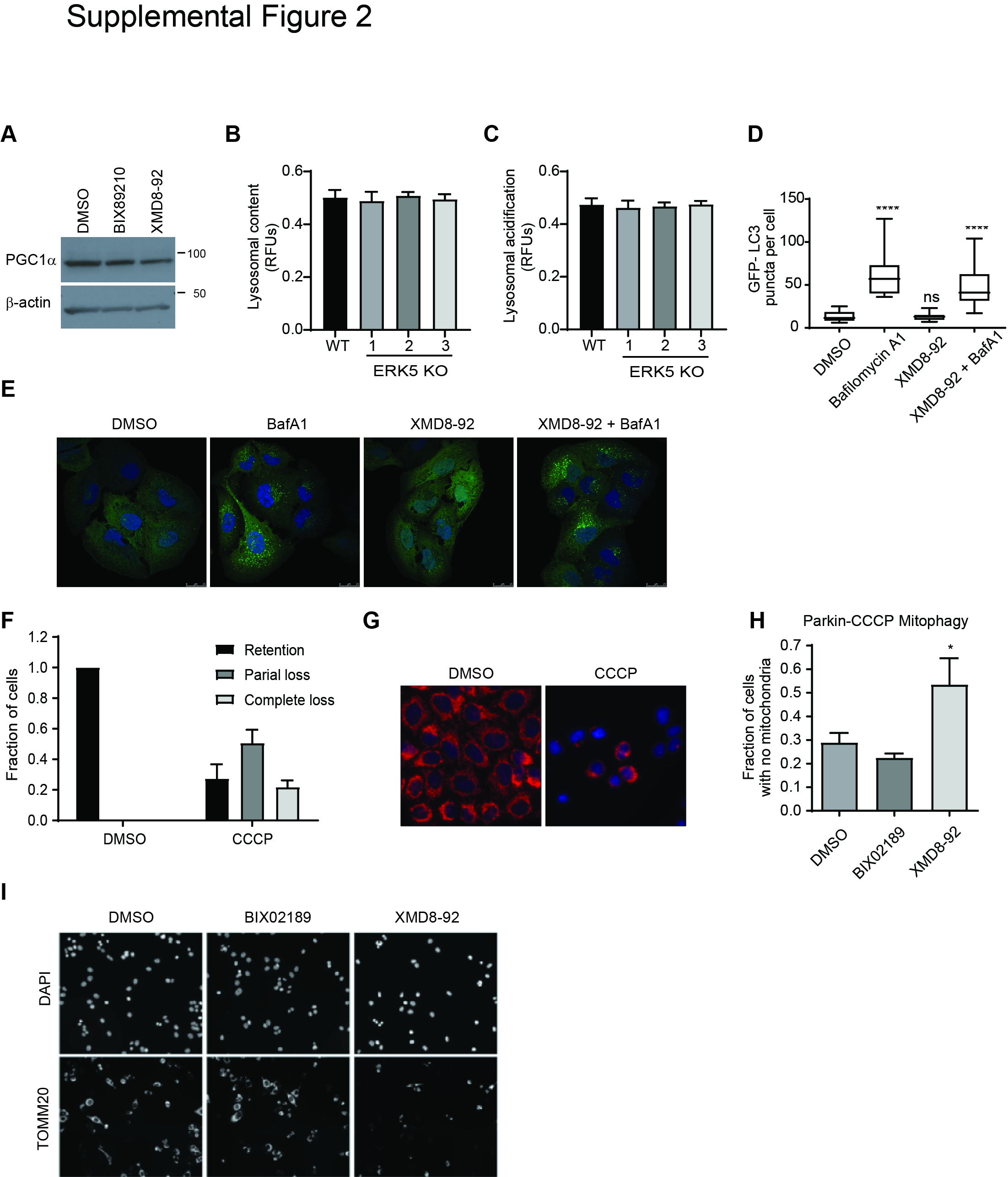

Supplement: Supplementary file 3 — Supplementary Figure S2 [file 41420_2020_342_MOESM3_ESM.tif]

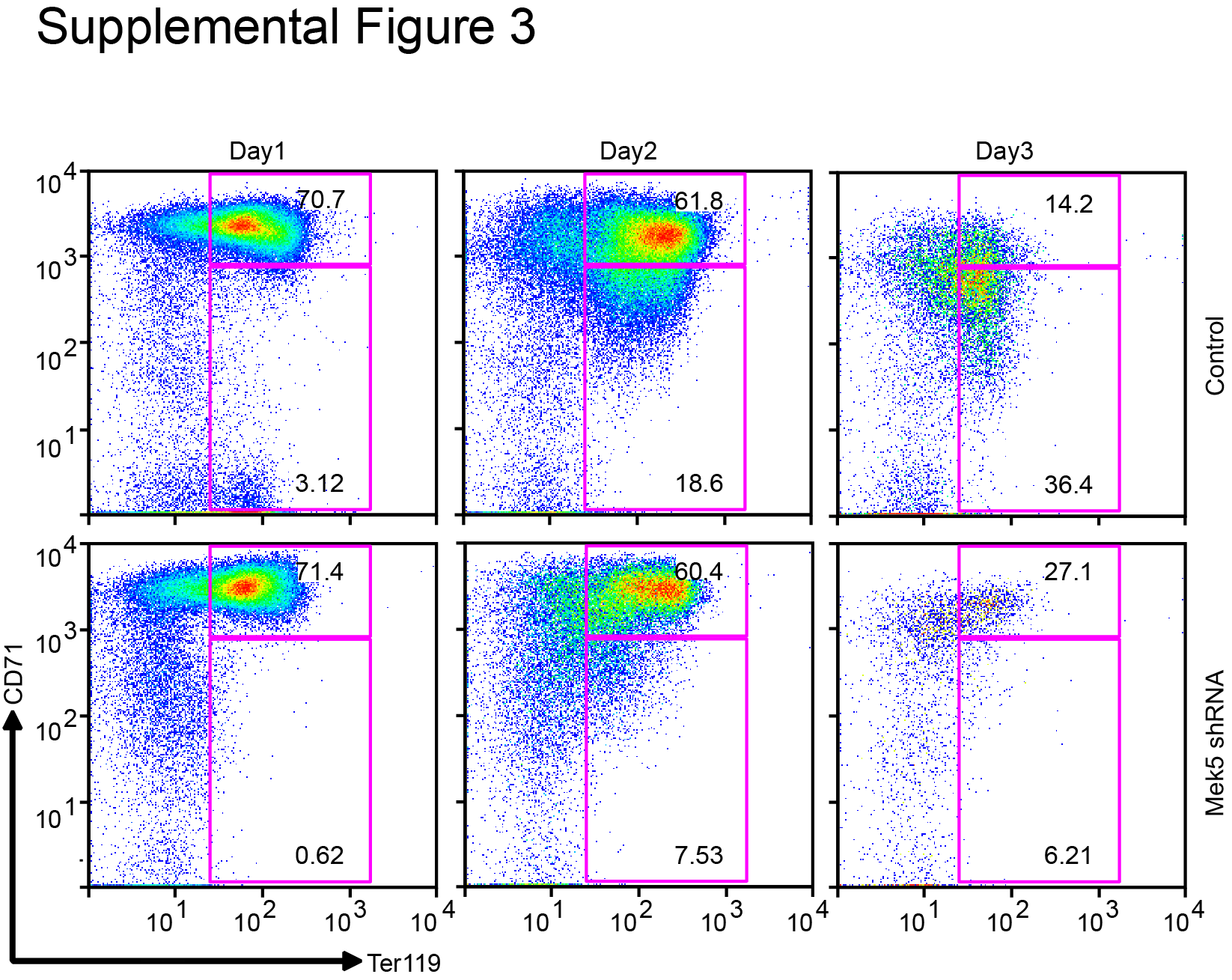

Supplement: Supplementary file 4 — Supplementary Figure S3 [file 41420_2020_342_MOESM4_ESM.tif]
